# 4-Cyano­pyridinium dihydrogen phosphate–isonicotinonitrile–phospho­ric acid (1/1/1)

**DOI:** 10.1107/S1600536812020430

**Published:** 2012-05-12

**Authors:** Ying-Chun Wang

**Affiliations:** a College of Chemistry and Chemical Engineering, Southeast University, Nanjing 210096, People’s Republic of China

## Abstract

The asymmetric unit of the title compound, C_6_H_5_N_2_^+^·H_2_PO_4_^−^·C_6_H_4_N_2_·H_3_PO_4_, contains one 4-cyano­pyridinium cation, one H_2_PO_4_^−^ anion, one independent isonicotinonitrile mol­ecule and one independent H_3_PO_4_ mol­ecule. The dihedral angle between the mean planes of the separate protonated and unprotonated pyridine rings is 9.93 (8)°. In the crystal, N—H⋯O and O—H⋯N hydrogen bonds and weak C—H⋯O and C—H⋯N inter­molecular inter­actions connect the organic mol­ecules into a two-dimensional network parallel to the *ac* plane. O—H⋯O hydrogen-bonding inter­actions involving the H_2_PO_4_^−^ anions and H_3_PO_4_ mol­ecules provide additional support from the inorganic groups Weak π–π stacking inter­actions between the pyridine rings of neighbouring organic mol­ecules [centroid–centroid distances = 3.711 (4) and 3.784 (2) Å] further link the layers into a three-dimensional network.

## Related literature

For the properties of related compounds, see: Chen *et al.* (2001 ▶); Huang *et al.* (1999 ▶); Zhang *et al.* (2001 ▶). For related structures, see: Wang *et al.* (2002 ▶); Xue *et al.* (2002 ▶); Ye *et al.* (2008 ▶).
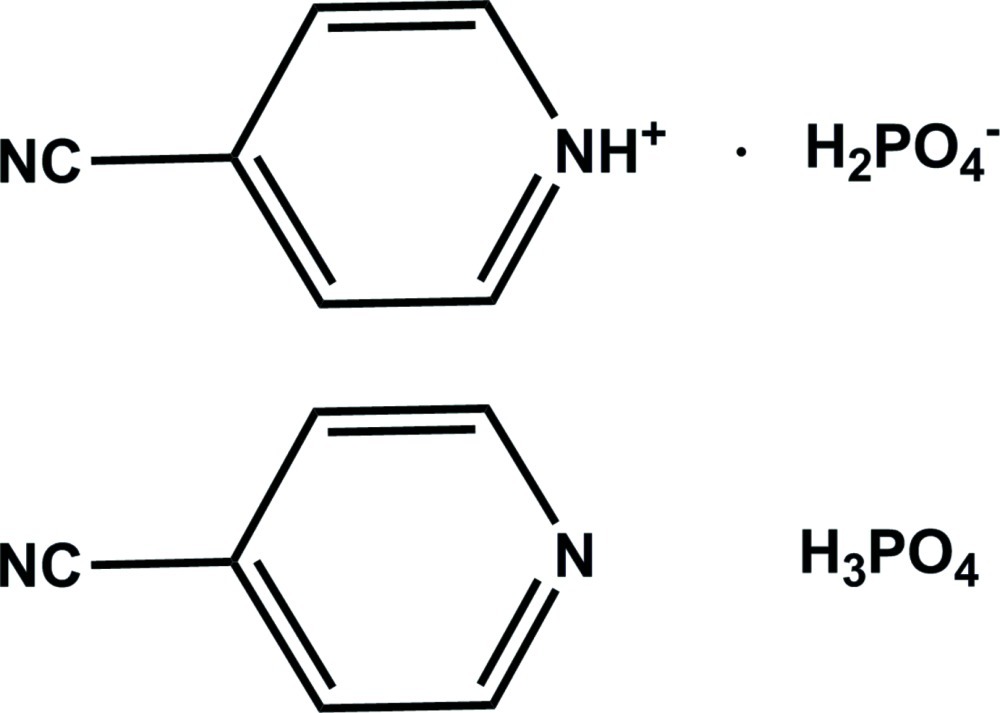


## Experimental

### 

#### Crystal data

C_6_H_5_N_2_^+^·H_2_O_4_P^−^·C_6_H_4_N_2_·H_3_O_4_P
*M* *_r_* = 404.21
Triclinic, 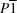

*a* = 8.1040 (5) Å
*b* = 8.8872 (9) Å
*c* = 12.1606 (8) Å
α = 81.491 (1)°
β = 82.009 (1)°
γ = 79.133 (1)°
*V* = 845.07 (11) Å^3^
*Z* = 2
Mo *K*α radiation
μ = 0.31 mm^−1^
*T* = 173 K
0.10 × 0.05 × 0.05 mm

#### Data collection

Rigaku Mercury2 diffractometer
Absorption correction: multi-scan (*CrystalClear*; Rigaku, 2005 ▶) *T*_min_ = 0.910, *T*_max_ = 1.000
8963 measured reflections
3798 independent reflections
3306 reflections with *I* > 2σ(*I*)
*R*_int_ = 0.023

#### Refinement

*R*[*F* ^2^ > 2σ(*F* ^2^)] = 0.030
*wR*(*F* ^2^) = 0.096
*S* = 1.14
3798 reflections
235 parameters
6 restraints
H-atom parameters constrained
Δρ_max_ = 0.34 e Å^−3^
Δρ_min_ = −0.40 e Å^−3^

### 

Data collection: *CrystalClear* (Rigaku, 2005 ▶); cell refinement: *CrystalClear*; data reduction: *CrystalClear*; program(s) used to solve structure: *SHELXS97* (Sheldrick, 2008 ▶); program(s) used to refine structure: *SHELXL97* (Sheldrick, 2008 ▶); molecular graphics: *SHELXTL* (Sheldrick, 2008 ▶); software used to prepare material for publication: *SHELXTL*.

## Supplementary Material

Crystal structure: contains datablock(s) I, global. DOI: 10.1107/S1600536812020430/jj2136sup1.cif

Structure factors: contains datablock(s) I. DOI: 10.1107/S1600536812020430/jj2136Isup2.hkl

Supplementary material file. DOI: 10.1107/S1600536812020430/jj2136Isup3.cml

Additional supplementary materials:  crystallographic information; 3D view; checkCIF report

## Figures and Tables

**Table 1:** Hydrogen-bond geometry (Å, °)

*D*—H⋯*A*	*D*—H	H⋯*A*	*D*⋯*A*	*D*—H⋯*A*
O2—H2⋯O5^i^	0.82	1.75	2.5576 (14)	169
O4—H4⋯O3^ii^	0.82	1.74	2.5611 (14)	176
O6—H6⋯N1^iii^	0.82	1.86	2.6749 (17)	178
O7—H7⋯O1^iv^	0.82	1.70	2.5150 (15)	173
O8—H8⋯O3^ii^	0.82	1.76	2.5795 (15)	177
N3—H3⋯O1	0.90	1.77	2.6466 (16)	162
C1—H1*A*⋯O2^v^	0.95	2.44	3.2549 (19)	144
C8—H8*A*⋯N2^vi^	0.95	2.51	3.273 (2)	138
C10—H10*A*⋯O7^vii^	0.95	2.31	3.1631 (19)	149
C11—H11*A*⋯O1^v^	0.95	2.52	3.3321 (19)	144

Symmetry codes: (i) 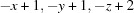; (ii) 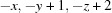; (iii) 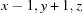; (iv) 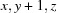; (v) 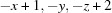; (vi) 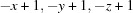; (vii) 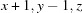.

**Fig. 1.**
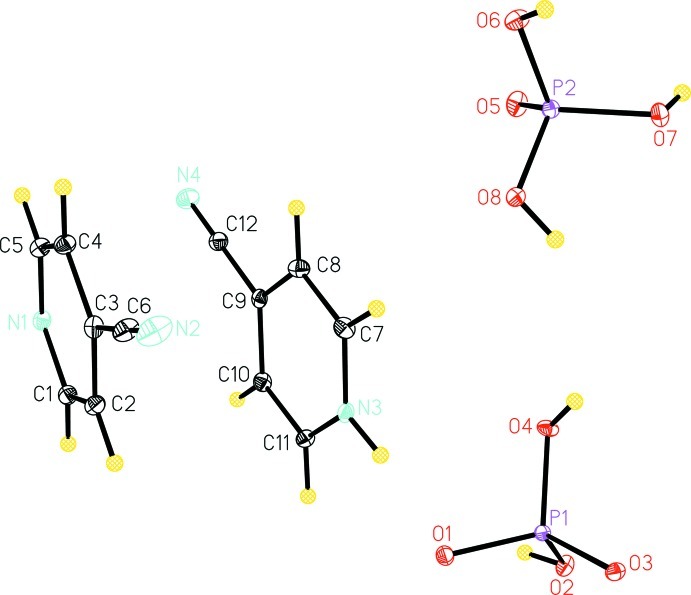
Molecular structure of the title compound showing the atom labeling scheme and 50% probability displacement ellipsoids for one cation-anion unit and bimolecular unit in the asymmetric unit.

**Fig. 2.**
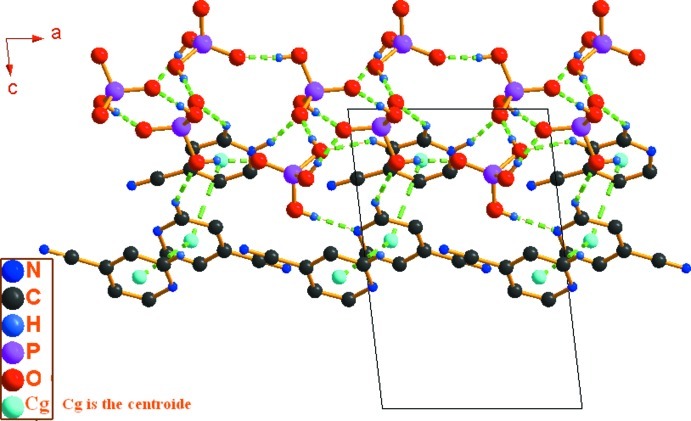
Crystal packing of the title compound viewed along the b axis showing O—H···O, O—H···N, hydrogen bonds (dotted lines), weak C—H···O, C—H···N intermolecular interactions (dotted lines) and weak π—π stacking interactions (dashed lines).

**Fig. 3.**
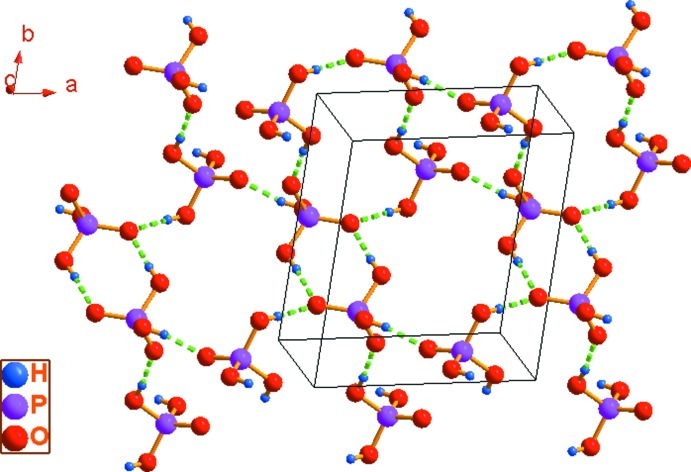
Crystal packing of the title compound viewed along the c axis showing the O—H···O hydrogen bonds (dotted line).

## supplementary crystallographic information

### Comment

Simple organic salts containing strong intrermolecular H-bonds have attracted attention as materials which display ferroelectric-paraelectric phase transitions (Chen *et al.*, 2001; Huang, *et al.* 1999; Zhang, *et al.* 2001). In an effort to obtain phase transition crystals of organic salts, various organic molecules have been studied with a series of new crystal materials (Wang *et al.*, 2002; Xue, *et al.* 2002; Ye *et al.*, 2008). Herewith, we present the synthesis and crystal structure of the title compound, C_6_H_5_N_2_^+^.H_2_PO_4_^-^.C_6_H_4_N_2_.H_3_PO_4_,(I).

The asymmetric unit of (I) is comprised of one 4-cyanopyridinium cation, one H_2_PO_4_^-^ anion, one independent isonicotinonitrile molecule and one independent H_3_PO_4_ molecule (Fig. 1). The two separate pyridine rings in the asymmetric unit are almost planar with the largest deviation from the least-squares plane being 0.001 (1) Å and 0.003 (1) Å, respectively. The dihedral angle between the mean planes of the two separate pyridine rings is 9.93 (8)°. Bond lengths and angles in each of these units are in normal ranges.

In the crystal N—H···O and O—H···N hydrogen bonds and weak C—H···O and C—H···N intermolecular interactions bring the organic molecules into a 2D network (Fig. 2). Also, O—H···O hydrogen bonding interactions involving the H_2_PO_4_^-^ anions and H_3_PO_4_ molecules provide additional support for the 2D network from the inorganic groups (Table 1, Fig. 3). In addition, weak π–π stacking interactions between the pyridine rings of neighbouring organic molecules further link the layers into a 3D network (Cg1···Cg2 = 3.711 (4) Å and Cg1···Cg2 = 3.784 (2) Å, where Cg1 and Cg2 are the centroids of the pyridine rings, N1/C1/C2/C3/C4/C5 and N3/C7/C8/C9/C10/C11, respectively).

### Experimental

Isonicotinonitrile (10 mmol and stirred at 60°C for 2 h. The precipitate was then filtrated. Colourless crystals suitable for X-ray diffraction were obtained by slow evaporation of the solution.

### Refinement

H2, H3, H4, H6 and H8 were refined freely. In the last stages of the refinement these atoms were restrained with N3—H3 = 0.90 (2)Å and O2—H2, O4—H4, O6–H6, O8—H8 all = 0.82 (2)Å with *U_iso_*(H) = 1.2*U_eq_*(N) and *U_iso_*(H)=1.5*U_eq_*(O). All the remaining H atoms attached to C atoms were placed in calculated positions and then refined using the riding model with C—H lengths of 0.95 Å (CH). The isotropic displcement parameers for these atoms were set to 1.2 (CH) times *U*_eq_ of the parent atom.

### Crystal data

**Table:**

C_6_H_5_N_2_^+^·H_2_O_4_P^−^·C_6_H_4_N_2_·H_3_O_4_P	*Z* = 2
*M**_r_* = 404.21	*F*(000) = 416
Triclinic, *P*1	*D*_x_ = 1.589 Mg m^−^^3^
Hall symbol: -P 1	Mo *K*α radiation, λ = 0.71073 Å
*a* = 8.1040 (5) Å	Cell parameters from 3798 reflections
*b* = 8.8872 (9) Å	θ = 2.6–27.5°
*c* = 12.1606 (8) Å	µ = 0.31 mm^−^^1^
α = 81.491 (1)°	*T* = 173 K
β = 82.009 (1)°	Block, colorless
γ = 79.133 (1)°	0.10 × 0.05 × 0.05 mm
*V* = 845.07 (11) Å^3^	

### Data collection

**Table:**

Rigaku Mercury2 diffractometer	3798 independent reflections
Radiation source: fine-focus sealed tube	3306 reflections with *I* > 2σ(*I*)
Graphite monochromator	*R*_int_ = 0.023
Detector resolution: 13.6612 pixels mm^-1^	θ_max_ = 27.5°, θ_min_ = 2.6°
CCD profile fitting scans	*h* = −10→10
Absorption correction: multi-scan (*CrystalClear*; Rigaku, 2005)	*k* = −11→11
*T*_min_ = 0.910, *T*_max_ = 1.000	*l* = −15→15
8963 measured reflections	

### Refinement

**Table:**

Refinement on *F*^2^	Primary atom site location: structure-invariant direct methods
Least-squares matrix: full	Secondary atom site location: difference Fourier map
*R*[*F*^2^ > 2σ(*F*^2^)] = 0.030	Hydrogen site location: inferred from neighbouring sites
*wR*(*F*^2^) = 0.096	H-atom parameters constrained
*S* = 1.14	*w* = 1/[σ^2^(*F*_o_^2^) + (0.0597*P*)^2^] where *P* = (*F*_o_^2^ + 2*F*_c_^2^)/3
3798 reflections	(Δ/σ)_max_ = 0.001
235 parameters	Δρ_max_ = 0.34 e Å^−^^3^
6 restraints	Δρ_min_ = −0.40 e Å^−^^3^

### Special details

**Table:**

Geometry. All esds (except the esd in the dihedral angle between two l.s. planes) are estimated using the full covariance matrix. The cell esds are taken into account individually in the estimation of esds in distances, angles and torsion angles; correlations between esds in cell parameters are only used when they are defined by crystal symmetry. An approximate (isotropic) treatment of cell esds is used for estimating esds involving l.s. planes.
Refinement. Refinement of *F*^2^ against ALL reflections. The weighted *R*-factor *wR* and goodness of fit *S* are based on *F*^2^, conventional *R*-factors *R* are based on *F*, with *F* set to zero for negative *F*^2^. The threshold expression of *F*^2^ > σ(*F*^2^) is used only for calculating *R*-factors(gt) *etc*. and is not relevant to the choice of reflections for refinement. *R*-factors based on *F*^2^ are statistically about twice as large as those based on *F*, and *R*- factors based on ALL data will be even larger.

### Fractional atomic coordinates and isotropic or equivalent isotropic displacement parameters (Å^2^)

**Table:**

	*x*	*y*	*z*	*U*_iso_*/*U*_eq_	
N1	1.02100 (16)	0.12068 (15)	0.59294 (11)	0.0141 (3)	
N2	0.39938 (18)	0.32104 (18)	0.46853 (13)	0.0273 (4)	
C4	0.83067 (19)	0.30110 (18)	0.48280 (13)	0.0158 (3)	
H4A	0.8118	0.3903	0.4294	0.019*	
C5	0.98861 (19)	0.24689 (18)	0.51769 (13)	0.0155 (3)	
H5A	1.0784	0.3010	0.4873	0.019*	
C6	0.5317 (2)	0.27525 (19)	0.49470 (14)	0.0188 (3)	
C2	0.73050 (19)	0.09074 (18)	0.60593 (13)	0.0152 (3)	
H2A	0.6429	0.0347	0.6378	0.018*	
C3	0.69979 (19)	0.22094 (18)	0.52833 (13)	0.0142 (3)	
C1	0.89403 (19)	0.04504 (18)	0.63541 (13)	0.0146 (3)	
H1A	0.9166	−0.0442	0.6883	0.018*	
N3	0.50667 (15)	0.28063 (14)	0.85938 (11)	0.0138 (3)	
H3	0.4049	0.2582	0.8897	0.017*	
N4	1.12529 (17)	0.40818 (17)	0.74487 (12)	0.0216 (3)	
C11	0.63720 (19)	0.18015 (18)	0.89738 (13)	0.0146 (3)	
H11A	0.6178	0.0886	0.9447	0.017*	
C8	0.6851 (2)	0.44610 (18)	0.75916 (13)	0.0158 (3)	
H8A	0.7008	0.5377	0.7107	0.019*	
C9	0.82275 (18)	0.34390 (17)	0.79861 (12)	0.0128 (3)	
C10	0.79957 (19)	0.20896 (18)	0.86819 (13)	0.0149 (3)	
H10A	0.8932	0.1385	0.8949	0.018*	
C7	0.52563 (19)	0.41132 (18)	0.79207 (13)	0.0162 (3)	
H7A	0.4294	0.4798	0.7671	0.019*	
C12	0.9921 (2)	0.37991 (18)	0.76837 (13)	0.0158 (3)	
P1	0.15691 (4)	0.29108 (4)	1.05923 (3)	0.00975 (11)	
O1	0.24327 (13)	0.17698 (12)	0.97909 (9)	0.0140 (2)	
O2	0.21239 (13)	0.23350 (12)	1.17856 (9)	0.0145 (2)	
H2	0.3149	0.2034	1.1716	0.022*	
O3	−0.03370 (12)	0.32218 (12)	1.06993 (9)	0.0131 (2)	
O4	0.22876 (13)	0.44436 (12)	1.01948 (9)	0.0140 (2)	
H4	0.1635	0.5162	0.9902	0.021*	
P2	0.31115 (5)	0.91637 (4)	0.78249 (3)	0.01113 (11)	
O5	0.47632 (13)	0.89331 (13)	0.82709 (9)	0.0184 (3)	
O6	0.32346 (13)	1.01043 (13)	0.66430 (9)	0.0163 (2)	
H6	0.2317	1.0444	0.6412	0.024*	
O7	0.16276 (13)	1.00119 (12)	0.85896 (9)	0.0153 (2)	
H7	0.1963	1.0539	0.8984	0.023*	
O8	0.24974 (14)	0.76550 (12)	0.76809 (9)	0.0167 (2)	
H8	0.1813	0.7401	0.8206	0.025*	

### Atomic displacement parameters (Å^2^)

**Table:**

	*U*^11^	*U*^22^	*U*^33^	*U*^12^	*U*^13^	*U*^23^
N1	0.0147 (6)	0.0160 (6)	0.0114 (7)	−0.0017 (5)	−0.0025 (5)	−0.0017 (5)
N2	0.0158 (7)	0.0357 (9)	0.0274 (9)	−0.0039 (6)	−0.0062 (6)	0.0085 (7)
C4	0.0170 (7)	0.0155 (7)	0.0138 (8)	−0.0025 (6)	−0.0025 (6)	0.0015 (6)
C5	0.0148 (7)	0.0173 (8)	0.0143 (8)	−0.0042 (6)	−0.0015 (6)	−0.0006 (6)
C6	0.0162 (8)	0.0215 (8)	0.0173 (8)	−0.0039 (6)	−0.0015 (6)	0.0027 (6)
C2	0.0154 (7)	0.0172 (8)	0.0134 (8)	−0.0046 (6)	−0.0009 (6)	−0.0013 (6)
C3	0.0129 (7)	0.0169 (7)	0.0128 (8)	−0.0006 (6)	−0.0032 (6)	−0.0029 (6)
C1	0.0175 (8)	0.0144 (7)	0.0113 (7)	−0.0017 (6)	−0.0024 (6)	−0.0006 (6)
N3	0.0107 (6)	0.0164 (6)	0.0145 (7)	−0.0030 (5)	0.0010 (5)	−0.0043 (5)
N4	0.0176 (7)	0.0241 (8)	0.0239 (8)	−0.0074 (6)	−0.0024 (6)	−0.0010 (6)
C11	0.0155 (7)	0.0148 (7)	0.0130 (8)	−0.0034 (6)	−0.0005 (6)	−0.0004 (6)
C8	0.0185 (8)	0.0148 (7)	0.0149 (8)	−0.0049 (6)	−0.0039 (6)	0.0000 (6)
C9	0.0131 (7)	0.0159 (7)	0.0111 (7)	−0.0038 (6)	−0.0012 (6)	−0.0057 (6)
C10	0.0131 (7)	0.0147 (7)	0.0160 (8)	0.0004 (6)	−0.0025 (6)	−0.0013 (6)
C7	0.0147 (7)	0.0155 (7)	0.0181 (8)	−0.0001 (6)	−0.0053 (6)	−0.0013 (6)
C12	0.0178 (8)	0.0167 (8)	0.0136 (8)	−0.0044 (6)	−0.0023 (6)	−0.0019 (6)
P1	0.00801 (19)	0.00916 (19)	0.0115 (2)	−0.00048 (14)	−0.00172 (14)	−0.00001 (14)
O1	0.0128 (5)	0.0137 (5)	0.0159 (6)	−0.0019 (4)	−0.0008 (4)	−0.0043 (4)
O2	0.0110 (5)	0.0181 (6)	0.0123 (6)	0.0013 (4)	−0.0024 (4)	0.0014 (4)
O3	0.0088 (5)	0.0123 (5)	0.0169 (6)	−0.0009 (4)	−0.0020 (4)	0.0019 (4)
O4	0.0108 (5)	0.0093 (5)	0.0213 (6)	−0.0010 (4)	−0.0052 (4)	0.0021 (4)
P2	0.00841 (19)	0.0120 (2)	0.0126 (2)	−0.00073 (14)	−0.00240 (14)	−0.00054 (15)
O5	0.0098 (5)	0.0251 (6)	0.0198 (6)	0.0011 (4)	−0.0054 (4)	−0.0024 (5)
O6	0.0110 (5)	0.0196 (6)	0.0164 (6)	−0.0024 (4)	−0.0037 (4)	0.0049 (4)
O7	0.0109 (5)	0.0162 (5)	0.0206 (6)	−0.0022 (4)	−0.0016 (4)	−0.0082 (4)
O8	0.0186 (6)	0.0138 (5)	0.0173 (6)	−0.0051 (4)	0.0047 (4)	−0.0037 (4)

### Geometric parameters (Å, º)

**Table:**

N1—C1	1.337 (2)	C8—C9	1.392 (2)
N1—C5	1.3477 (19)	C8—H8A	0.9500
N2—C6	1.144 (2)	C9—C10	1.389 (2)
C4—C5	1.381 (2)	C9—C12	1.453 (2)
C4—C3	1.394 (2)	C10—H10A	0.9500
C4—H4A	0.9500	C7—H7A	0.9500
C5—H5A	0.9500	P1—O3	1.5077 (10)
C6—C3	1.450 (2)	P1—O1	1.5176 (11)
C2—C3	1.387 (2)	P1—O2	1.5635 (11)
C2—C1	1.391 (2)	P1—O4	1.5666 (11)
C2—H2A	0.9500	O2—H2	0.8195
C1—H1A	0.9500	O4—H4	0.8198
N3—C11	1.3370 (19)	P2—O5	1.4811 (11)
N3—C7	1.339 (2)	P2—O6	1.5526 (11)
N3—H3	0.9008	P2—O8	1.5560 (11)
N4—C12	1.142 (2)	P2—O7	1.5601 (11)
C11—C10	1.376 (2)	O6—H6	0.8196
C11—H11A	0.9500	O7—H7	0.8208
C8—C7	1.377 (2)	O8—H8	0.8198
			
C1—N1—C5	118.22 (13)	C10—C9—C12	119.62 (14)
C5—C4—C3	117.97 (14)	C8—C9—C12	119.72 (14)
C5—C4—H4A	121.0	C11—C10—C9	118.16 (14)
C3—C4—H4A	121.0	C11—C10—H10A	120.9
N1—C5—C4	122.96 (14)	C9—C10—H10A	120.9
N1—C5—H5A	118.5	N3—C7—C8	119.76 (14)
C4—C5—H5A	118.5	N3—C7—H7A	120.1
N2—C6—C3	178.62 (18)	C8—C7—H7A	120.1
C3—C2—C1	117.73 (14)	N4—C12—C9	179.83 (17)
C3—C2—H2A	121.1	O3—P1—O1	115.74 (6)
C1—C2—H2A	121.1	O3—P1—O2	108.01 (6)
C2—C3—C4	119.98 (14)	O1—P1—O2	109.65 (6)
C2—C3—C6	120.42 (14)	O3—P1—O4	110.60 (6)
C4—C3—C6	119.60 (14)	O1—P1—O4	106.72 (6)
N1—C1—C2	123.16 (14)	O2—P1—O4	105.66 (6)
N1—C1—H1A	118.4	P1—O2—H2	107.9
C2—C1—H1A	118.4	P1—O4—H4	115.8
C11—N3—C7	122.80 (13)	O5—P2—O6	109.29 (6)
C11—N3—H3	113.8	O5—P2—O8	115.10 (6)
C7—N3—H3	123.0	O6—P2—O8	105.90 (6)
N3—C11—C10	120.21 (14)	O5—P2—O7	113.15 (6)
N3—C11—H11A	119.9	O6—P2—O7	109.13 (6)
C10—C11—H11A	119.9	O8—P2—O7	103.84 (6)
C7—C8—C9	118.43 (15)	P2—O6—H6	114.0
C7—C8—H8A	120.8	P2—O7—H7	111.9
C9—C8—H8A	120.8	P2—O8—H8	112.0
C10—C9—C8	120.65 (14)		
			
C1—N1—C5—C4	0.0 (2)	C7—N3—C11—C10	0.3 (2)
C3—C4—C5—N1	0.2 (2)	C7—C8—C9—C10	0.9 (2)
C1—C2—C3—C4	0.0 (2)	C7—C8—C9—C12	−177.97 (14)
C1—C2—C3—C6	−179.54 (14)	N3—C11—C10—C9	−0.2 (2)
C5—C4—C3—C2	−0.2 (2)	C8—C9—C10—C11	−0.5 (2)
C5—C4—C3—C6	179.34 (14)	C12—C9—C10—C11	178.46 (14)
C5—N1—C1—C2	−0.2 (2)	C11—N3—C7—C8	0.2 (2)
C3—C2—C1—N1	0.2 (2)	C9—C8—C7—N3	−0.8 (2)

### Hydrogen-bond geometry (Å, º)

**Table:**

*D*—H···*A*	*D*—H	H···*A*	*D*···*A*	*D*—H···*A*
O2—H2···O5^i^	0.82	1.75	2.5576 (14)	169
O4—H4···O3^ii^	0.82	1.74	2.5611 (14)	176
O6—H6···N1^iii^	0.82	1.86	2.6749 (17)	178
O7—H7···O1^iv^	0.82	1.70	2.5150 (15)	173
O8—H8···O3^ii^	0.82	1.76	2.5795 (15)	177
N3—H3···O1	0.90	1.77	2.6466 (16)	162
C1—H1*A*···O2^v^	0.95	2.44	3.2549 (19)	144
C8—H8*A*···N2^vi^	0.95	2.51	3.273 (2)	138
C10—H10*A*···O7^vii^	0.95	2.31	3.1631 (19)	149
C11—H11*A*···O1^v^	0.95	2.52	3.3321 (19)	144

Symmetry codes: (i) −*x*+1, −*y*+1, −*z*+2; (ii) −*x*, −*y*+1, −*z*+2; (iii) *x*−1, *y*+1, *z*; (iv) *x*, *y*+1, *z*; (v) −*x*+1, −*y*, −*z*+2; (vi) −*x*+1, −*y*+1, −*z*+1; (vii) *x*+1, *y*−1, *z*.
